# A Quantitative Study Exploring and Comparing Key Factors in Medication Management in the Irish Healthcare Setting

**DOI:** 10.1111/hex.70256

**Published:** 2025-04-12

**Authors:** Bernadine O'Donovan, Ciara Kirke, Muriel Pate, Sheena McHugh, Kathleen Bennett, Caitríona Cahir

**Affiliations:** ^1^ Data Science Centre, School of Population Health RCSI University of Medicine and Health Sciences Dublin Ireland; ^2^ Medication Safety, Quality Improvement Division Health Service Executive (HSE) Dublin Ireland; ^3^ School of Public Health University College Cork Cork Ireland

**Keywords:** BMQ, medication beliefs, medication management, patient knowledge, patient self‐efficacy, quantitative research, shared decision‐making

## Abstract

**Background:**

Shared decision‐making in the context of medication management has been shown to be contingent on information‐seeking behaviours such as patient knowledge, self‐efficacy and engagement.

**Objective:**

The aim of this study was to: (i) compare differences in perceptions of patients' knowledge, capabilities and engagement across healthcare professionals (HCPs) and patients and family caregivers and (ii) investigate associations between these factors and patients' medication beliefs using a cross‐sectional survey study of patients, family caregivers and community and hospital HCPs in Ireland.

**Methods:**

Two cross‐sectional surveys measuring key factors in medication management were distributed to patients and family caregivers taking three or more medicines and HCPs involved in medicines management. *χ* ^2^ tests were used to investigate differences between HCPs and patients and family caregivers. Multivariable linear regression with adjustment for the socio‐demographic covariates was used to examine key factors in medication management and beliefs about medicine (BMQ‐General) in patients and family caregivers.

**Results:**

Overall, 636 responses were received; patients and family caregivers (*N* = 134, 21%), community (*N* = 313, 49%) and hospital HCPs (*N* = 189, 30%). A higher proportion of patients and family caregivers self‐reported as ‘knowledgeable’ about medications (*N* = 76; 56.7%) than community (*N* = 75, 24%) and hospital HCPs (*N* = 44, 23.3%) (*p* < 0.01). The majority of patients and family caregivers were ‘fairly/very confident’ they could maintain an accurate medication list without assistance (*N* = 78; 58.2%), compared to the majority of the community (*N* = 213, 68.1%) and hospital HCPs (*N* = 114, 60.3%) who were ‘not at all/somewhat confident’ (*p* < 0.01.) These patients and family caregivers also had significantly lower overall beliefs in medication harm (*β* = −1.23, 95% CI: −2.34, −0.13). Patient and family caregivers who asked HCPs about their medication frequently (> 7 times per year) had higher overall beliefs in medication overuse (*β* = 1.88, 95% CI: 0.06, 3.69) and medication harm (*β* = 2.65, 95% CI: 1.10, 4.20), compared to those who never asked.

**Conclusion:**

There was divergence between HCPs and patients and family caregivers in their assessments of patients' medication knowledge and capabilities. Engagement between HCPs and patients around medication should be purposeful rather than frequent, to alleviate fears about overuse and harm.

**Patient or Public Contribution:**

The patient and family caregiver survey was developed in partnership with members of the Patient and Public Involvement (PPI) group. Feedback was provided by the group to increase accessibility of survey and maximise distribution. In addition, the survey was piloted among members of the public involved in medication management.

## 1 Introduction

Polypharmacy and multimorbidity are associated with a greater risk of medication‐related harm and a higher number of healthcare transitions with associated medication‐related risks [1, 2]. Healthcare intervention can involve multiple interactions over time, as well as multiple transfers of information about medicines across healthcare systems (e.g., primary care to hospital, outpatient appointments) [3]. A key aspect of patient‐centred care for all patients, including those with multimorbidity and polypharmacy, is shared or supported decision‐making (SDM) [4]. Collaborative decision‐making in healthcare is a pooling of resources by groups to achieve their health goals [5]. SDM is a more developed collaborative process, whereby the healthcare professional (HCP) works in partnership with their patient to support them in making decisions about their care [6].

SDM consists of a number of stages, including: (i) provision of evidence‐based information about options, outcomes and uncertainties to the patient; (ii) discussion of treatment options in relation to the patient's own wishes and values; (iii) discussion of implications of the patient's chosen course of action or inaction, including the risk and benefits associated with that action and (iv) recording and implementing patient's informed preferences [7, 8].

UK, US, and Australian studies in a variety of populations have demonstrated that involving patients in their care leads to improved adherence and health outcomes [9, 10, 11, 12]. Many patients prefer shared/supported clinician‐led decision‐making, where the practitioner makes decisions for the patient. SDM can empower patients, leading to higher‐quality medical decisions, enhanced therapeutic alliances and reductions in symptom severity [13, 14, 15, 16]. Shared or SDM between HCPs and patients has become an important—and often mandated—part of contemporary healthcare [17, 18]. In the United Kingdom, SDM is enshrined in the National Health Service (NHS) Constitution [19, 20]. In many European countries, Canada, Australia and the United States, there are guidelines, policies and health reform legislation that support SDM, and it is also strongly supported by patient organisations [21, 22, 23, 24, 25]. In Ireland, there are no clear specific guidelines to support SDM across the healthcare systems. However, there is a general acceptance of adopting a more inclusive, informed, patient‐centred model, which considers the patient's input, experience and decisions [26].

There is some evidence in Ireland that patients experience elements of SDM in their healthcare—including information sharing by HCPs, being listened to and involved in treatment, operations and procedure decisions. However, there has been limited research on the use of SDM in medication‐related decisions. Several barriers to the use of SDM in medication management have been identified in previous research. These include lack of familiarity among HCPs with the SDM process, time constraints within clinical practice, low levels of patient health literacy and self‐efficacy, lack of healthcare systems structures and limited support of medication reviews/discussions [27, 28, 29, 30]. There is evidence that HCPs do not generally discuss the implications of taking medications with their patients, particularly those with long‐term chronic conditions [23]. However, a higher SDM level has been shown to be significantly associated with a lower risk of polypharmacy in older patients [31, 32].

Research also suggests that SDM for patients is dependent on a few key factors—including patient knowledge (knowledge about treatment options), patient self‐efficacy (confidence in the ability to acquire knowledge and perceived capacity to influence the treatment decision) and level of engagement (in relation to medications) with HCPs [22, 23, 24]. These factors can be potential barriers to SDM for HCPs, but it is unclear if HCP perceptions and understanding of these patient‐level factors are different from those of patients and family caregivers. There is also limited understanding of the role of these factors in effective SDM, within the context of medication management. Previous US and European studies have established the role of medication beliefs in medication adherence [33, 34, 35, 36, 37]. There is some evidence that patients' beliefs about medication—perceived use of medication and concern about harm—can influence their motivation to participate in SDM. Further research is needed to understand the complex interplay between patient knowledge, self‐efficacy, engagement with HCPs and their medications beliefs, within the context of SDM [38, 39, 40].

The aim of this study was to: (i) examine differences in HCP perceptions of patients' and family caregivers' knowledge about medicines; the capabilities of patients and family caregivers to manage medicines and the level of engagement about medicines by HCPs compared to patients and family caregivers and (ii) investigate the association between these key SDM factors and the medication beliefs of patients and family caregivers using a cross‐sectional survey study of patients, family caregivers and community and hospital HCPs in Ireland.

## 2 Methods

The study is reported using the STrengthening the Reporting of OBservational studies in Epidemiology (STROBE) checklist [41].

### 2.1 Study Design, Setting, Participants and Survey Design

This was a cross‐sectional survey study of patients, family caregivers and community and hospital HCPs in Ireland. Participants were stratified by age, gender and region in Ireland. The patient sample was composed of adult patients, who were prescribed multiple medicines (3 or more medicines) and had at least one chronic long‐term illness. There was a separate sample of family caregivers, who provided unpaid, informal care and support to family members (prescribed 3 or more medicines). Community and hospital HCPs involved in medication management in Ireland, including General Practitioners (GPs), community pharmacists, community nurses, as well as hospital doctors, hospital pharmacists and hospital nurses were invited to participate. Two separate surveys, based on the findings from previous qualitative interviews, were developed—one for patients and family caregivers and one for HCPs [42]. The two surveys were developed and piloted with a Patient and Public Involvement (PPI) group and HCPs involved in medicines management. Feedback on the language, ease of use, clarity and structure of the surveys was solicited. Discussions with PPI members resulted in revisions to the surveys, which improved comprehension and ensured relevant information was collected.

### 2.2 Survey Instrument

The surveys were divided into three sections measuring key factors in medication management including: (i) patient and family caregivers' knowledge about medicines, (ii) patient and family caregivers' capabilities to manage medicines, (iii) patterns of engagement with patients and family caregivers about medicines and (iv) beliefs about medicines (see Supporting Information for survey questions). Two types of Likert scales were used—4‐point scales to determine levels of knowledge and capabilities and 5‐point scales to assess agreement with statements related to levels of engagement. Both groups, patients and family caregivers (Group 1) and community and hospital HCPs (Group 2), were asked to evaluate patients and family caregivers' knowledge about their medicines and their confidence in patients and family caregivers being capable of keeping an accurate medicine list. Group 1 were also asked to self‐report their confidence in their ability to ask for help with their medicine list, if needed. Both knowledge about medicines and confidence in capabilities were assessed on a 4‐point Likert scale (ranging from 1 = not knowledgeable to 4 = very knowledgeable and from 1 = not at all confident to 4 = very confident).

Information about patterns of engagement with medicines was also gathered. Group 2 were asked about the frequency of their engagement with medication lists, for example, how often they asked about lists or whether patients or family caregivers shared lists with them. Agreement with statements related to engagement was rated on a 5‐point Likert scale (1 = never; 5 = always). Group 1 were asked if they had engaged with any HCP about their medicines in the past 12 months. Both groups were also asked their opinion on the time required, during consultations to engage with people about their medicines ( < 10 min, ≥ 10 min and varying time).

Medication beliefs were measured using the BMQ general beliefs about medicines—this consists of the General‐overuse (Go) sub‐scale, assessing beliefs about the use of medicines by doctors (4 statements), and the General‐harm (Gh) sub‐scale, assessing beliefs about the degree to which medicines are perceived as harmful (4 statements) [43]. Each statement has a 5‐point Likert scale response, scored from 1 (strongly disagree) to 5 (strongly agree). A scale score is calculated by summing these scores, with higher scores (above the mid‐point) on the BMQ Go and BMQ Gh scales, indicating stronger beliefs about medicines overuse/harm [44]. Group 2 were asked to report their age, gender and clinical experience. Group 1 were asked to report their age and gender.

### 2.3 Data Collection

Invitation emails, participant information leaflets and links to the anonymised online surveys were distributed to HCPs, patients and family caregivers via relevant professional bodies (e.g., Irish College of General Practitioners and Pharmaceutical Society of Ireland), patient groups, social media and contacts within the research group. Reminder emails were sent to all professional organisations, invitations to participate were issued on social media, and the surveys were available online for 5 months (February–June 2022). Ethical approval was obtained from the RCSI research ethics committee (REC: 212601097), and all data collection and processing was in compliance with RCSI data protection policies, General Data Protection Regulation (GDPR) and Data Protection Bill 2018 (Data Protection Act) (Section 36(2)) (Health Research) Regulations 2018. A data management plan was created, and no IP address tracking was performed.

### 2.4 Data Analysis

Descriptive statistics, including means (standard deviation, SD), medians (inter‐quartile range, IQR) and frequencies and percentages (95% confidence intervals [CIs]), were calculated for all survey items. The method of distribution meant the response rate was not calculated. The *χ* ^2^ test of independence (*χ* ^2^)—with its degrees of freedom, sample size and *p* value—was used to investigate differences between the groups. Community and hospital HCPs were compared to patients and family caregivers in their perceptions of: (i) patients' and family caregivers' knowledge about medicines; (ii) confidence in patients' and family caregivers' abilities to maintain an accurate medication list and (iii) the time needed to engage with patients and family caregivers about medicines.

The associations between these three key factors in medication management: knowledge; confidence in capabilities; engagement about medicines and the BMQ Go sub‐scale and the Gh sub‐scale in the patient and family caregiver group were assessed using multivariable linear regression models. Adjustments for age and gender and robust standard errors (sandwich estimator) were used to control for mild violation of the homoscedasticity and normality distribution assumption in linear regression. Unadjusted and adjusted *β* coefficients and 95% CIs are presented. Data was analysed using Stata Version 17.0 (StataCorp, College Station, Texas, the United States). Significance at *p* < 0.05 was assumed. Incomplete surveys were dealt with using the conventional method of pairwise exclusion of missing data from analysis [45].

## 3 Results

Overall, 636 responses were received: patients and family caregivers (*N* = 134, 21%), community HCPs (*N* = 313, 49%) and hospital HCPs (*N* = 189, 30%) (Table 1). There were 87 patients and 47 family caregivers. More than half of all patients and family caregivers were female (*N* = 67, 63.2%); the majority were aged 25–54 years (*N* = 83, 78.3%) with approximately one‐fifth (21.7%) aged 55–74 years. Community HCPs were composed of GPs (*N* = 124, 40%), community pharmacists (*N* = 144, 46%) and community nurses (*N* = 45, 14%). Most were female (*N* = 152, 60.1%), aged 25–44 years (*N* = 180; 75.3%) and the majority had more than 10 years in practice (*N* = 191, 81.3%). Hospital HCPs included hospital doctors (*N* = 23, 12%), hospital pharmacists (*N* = 150, 79%) and hospital nurses (*N* = 16, 9%). The majority were female (*N* = 108, 82.4%) and aged 25–44 years (*N* = 104; 85.3%), with more than 10 years in practice (*N* = 93, 73.8%).

**Table 1:** Participant characteristics in the community and hospital HCPs and patient and family caregivers survey response groups.

	Community HCPs	Hospital HCPs	Patients and family caregivers
**Age**	*N* (%) (95% CI) (*N* = 239)	*N* (%) (95% CI) (*N* = 122)	*N* (%) (95% CI) (*N* = 106)
25–44 years	180 (75.3%; 69.4, 80.4)	74 (60.7%; 51.6, 69)	34 (32.1%; 23.8, 41.6)
45–54 years	34 (14.2%; 10.3, 19.3)	30 (24.6%; 17.7, 33.1)	49 (46.2%; 36.9, 55.8)
55–74 years	25 (10.5%; 7.2, 15.1)	18 (14.8%; 9.5, 22.2)	23 (21.7%; 14.8, 30.7)
**Gender**	*N* (%) (95% CI) (*N* = 253)	*N* (%) (95% CI) (*N* = 131)	*N* (%) (95% CI) (*N* = 106)
Female	152 (60.1; 34, 46.1)	108 (82.4; 74.9, 88)	67 (63.2; 53.5, 71.9)
Male	101 (39.9; 53.9, 66)	23 (17.6; 11.9, 25.1)	39 (36.8; 53.5, 71.9*)*
**Years qualified**	*N* (%) (95% CI) (*N* = 235)	*N* (%) (95% CI) (*N* = 126)	*N* (%)
< 5	13 (5.5; 3.3, 9.3)	11 (8.7; 4.9, 15.1)	N/A
5–10	31 (13.2; 9.4, 18.2)	22 (17.5; 11.7, 25.2)	N/A
11–15	46 (19.6; 15, 25.2)	27 (21.4; 15.1, 29.5)	N/A
16–20	86 (36.6; 30.7, 43)	19 (15.1; 9.8, 22.5)	N/A
21–25	35 (14.9; 10.9, 20.1)	25 (19.8; 13.7, 27.8)	N/A
> 25	24 (10.2; 6.9, 14.8)	22 (17.5; 11.7, 25.2)	N/A

Abbreviation: N/A = not applicable.

### 3.1 Key Factors in Medication Management

#### 3.1.1 Patient and Family Caregiver Knowledge About Medicines

A larger proportion of patients and family caregivers reported they were knowledgeable about their medications (*N* = 76, 56.7%; [95% CI: 48.1, 64.9]; see Table 2) compared to community and hospital HCPs (*N* = 75, 24%; [95% CI: 19.5, 29] and *N* = 44, 23.3%; [95% CI: 17.8, 29.9], respectively). There was a statistically significant difference between the groups (*χ* ^2^ = 54.24, df = 8, *p* < 0.01).

**Table 2:** Key factors in medication management in community and hospital HCPs and patients and family caregivers.

	Community HCPs (*N* = 313)	Hospital HCPs (*N* = 189)	Patients and caregivers (*N* = 134)
**Patient's knowledge of medications**	*N* (%) (95% CI)	*N* (%) (95% CI)	*N* (%) (95% CI)
Not very knowledgeable	238 (76.1; 71, 80.5)	145 (76.7; 70.1, 82.2)	58 (43.3; 35.1, 51.9)
Knowledgeable	75 (24; 19.5, 29.0)	44 (23.3; 17.8, 29.9)	76 (56.7; 48.1, 64.9)
**Confidence in the patient's ability to maintain an accurate medication list**	*N* (%) (95% CI)	*N* (%) (95% CI)	*N* (%) (95% CI)
Not at all/somewhat confident	213 (68.1; 62.7, 73)	114 (60.3; 53.1, 67.1)	56 (41.8; 33.7, 50.4)
Fairly confident/very confident	100 (32; 27, 37.3)	75 (39.7; 32.9, 46.9)	78 (58.2; 49.6, 66.3)
**Patient confidence in asking for help with medication list**	*N* (%) (95% CI)	*N* (%) (95% CI)	*N* (%) (95% CI)
Not at all/somewhat confident	N/A	N/A	42 (31.3; 24, 39.8
Fairly confident/very confident	N/A	N/A	92 (68.7; 60.2, 76)
**Engagement about medicines**	*N* (%) (95% CI)	*N* (%) (95% CI)	*N* (%) (95% CI)
HCPs asked patients about medication lists^#^			
Never	49 (15.7; 12, 20.1)	24 (12.7; 8.6, 18.3)	N/A
Sometimes	177 (56.5; 51, 62)	46 (24.3; 18.7, 31)	N/A
Often/always	87 (27.8; 23.1, 33)	119 (63; 55.8, 69.6)	N/A
Patients share medication lists with HCPs			
Never	67 (21.4; 17.2, 26.3)	24 (12.7; 8.6, 18.3)	N/A
Sometimes	159 (50.8; 45.3, 56.3)	56 (29.6; 23.5, 36.6)	N/A
Often/always	87 (27.8; 23.1, 33)	109 (57.7; 50.5, 64.6)	N/A
Patients asked HCPs about medications			
Never	N/A	N/A	33 (24.6; 18, 32.7)
1–2 times	N/A	N/A	60 (44.8; 36.5, 53.3)
3–6 times	N/A	N/A	22 (16.4; 11, 23.8)
> 7 times	N/A	N/A	19 (14.2; 9.2; 21.2)
**Time needed to engage**	*N* (%) (95% CI)	*N* (%) (95% CI)	*N* (%) (95% CI)
< 10 min	246 (78.6; 73.7, 82.8)	57 (36.5; 29.3, 44.4)	76 (63.3; 54.3, 71.5)
≥10 min	54 (17.3; 13.4, 21.9)	68 (43.6; 36, 51.5)	22 (18.3; 12.3, 26.4)
Varying time	13 (4.2; 2.4, 7)	31 (19.9; 14.3, 26.9)	22 (18.3; 12.3, 26.4)

#### 3.1.2 Patients' and Family Caregivers' Capabilities to Manage Medicines

The majority of patients and family caregivers were confident in their abilities to maintain an accurate medication list without assistance (*N* = 78, 58.2%; [95% CI: 49.6, 66.3]) or ask for help from HCPs if needed (*N* = 92, 68.7%; [95% CI: 60.2, 76.0]). This contrasts with HCPs who were not confident in the abilities of their patients or family caregivers (community HCPs *N* = 213, 68.1%; [95% CI: 62.7, 73]) and hospital HCPs (*N* = 114, 60.3%; [95% CI: 53.1; 67.1]). There was a statistically significant difference between the groups (*χ* ^2^ = 27.01, df = 8, *p* < 0.01).

#### 3.1.3 Engagement About Medicines by HCPs and Patients and Family Caregivers

Hospital HCPs frequently asked patients or family caregivers about medication lists (*N* = 119, 63%; [95% CI: 55.8%, 69.6%]), and a majority of patients and family caregivers often shared their list (*N* = 109, 57.7%; [95% CI: 50.5%, 64.6%]). Community HCPs did not frequently ask about lists (*N* = 177, 56.5%; [95% CI: 51%, 62%]), and patients and family caregivers did not frequently share their lists with them (*N* = 159, 50.8%; [95% CI: 45.3%, 56.3%]). Over a third of patients and family caregivers asked an HCP about their medicines, at least 1–2 times in the previous year (*N* = 60, 44.8%; [95% CI: 36.5%, 53.3%]). However, approximately a quarter (*N* = 33, 24.6%; [95% CI: 18%, 32.7%]) had not asked about medicines in the past year. The majority of patients and family caregivers (*N* = 76, 63.3%; [95% CI: 54.3%, 71.5%]) and community HCPs (*N* = 246, 78.6%; [95% CI: 73.7%, 82.8%]) considered less than 10 min was needed to engage with patients about their medicines. However, over a third of hospital HCPs (*N* = 68, 43.6%; [95% CI: 36%, 51.5%]) reported more time was needed ( ≥ 10 min). There was a statistically significant difference between the groups (*χ* ^2^ = 90.27, df = 8, *p* < 0.01).

#### 3.1.4 Medication Beliefs Among Patients and Family Caregivers

The majority of patient and family caregivers did not believe that medications were harmful (*N* = 98, 80%; [95% CI: 72.2, 86.5]; see Table 3). However, approximately a third of patients and family caregivers believed that medicines were overused by doctors (*N* = 34, 27.2%; [95% CI 20.1%, 35.7%]). The median BMQ Go score was 10.5 (IQR 9, 12); the median BMQ Gh score was 8 (IQR 5, 9); and the overall median BMQ score was 18 (IQR 15, 22).

**Table 3:** BMQ General‐overuse and General‐harm scale scores for patients and family caregivers.

General‐overuse (Go) scale	Agree/strongly agree *N* (%) (95% CI)	Neither agree/disagree *N* (%) (95% CI)	Disagree/strongly disagree *N* (%) (95% CI)
BMQ_1 Doctors use too many medicines	34 (27.2; 20.1, 35.7)	59 (47.2; 38.5, 56)	32 (25.6, 18.7, 34)
BMQ_4 Natural remedies are safer than medicines	3 (2.5; 0.8, 7.5)	38 (31.4; 23.7, 40.3)	80 (66.1; 57.2, 74.1)
BMQ_7 Doctors place too much trust in medicines	25 (20.8; 14.4, 29.1)	33 (27.5; 20.2, 36.2)	62 (51.7; 42.7, 60.5)
BMQ_8 If doctors had more time with patients, they would prescribe fewer medicines	38 (31.9; 24.1, 40.9)	31 (26.1; 18.9; 34.8)	50 (42; 33.4, 51.1)
**General harm (Gh) scale**			
BMQ_2 People who take medicines should stop their treatment for a while every now and again	5 (4.1; 1.2, 9.5)	25 (20.5; 14.2, 28.7)	92 (75.4; 66.9, 82.3)
BMQ_3 Most medicines are addictive	12 (9.8; 5.6, 16.6)	24 (19.7; 13.5, 27.8)	86 (70.5; 61.7, 78)
BMQ_5 Medicines do more harm than good	7 (5.7; 2.7, 11.6)	17 (13.9; 8.8, 21.4)	98 (80.; 72.2, 86.5)
BMQ_6 All medicines are poisons	9 (7.5; 3.9, 13.9)	9 (7.5; 3.9, 13.9)	102 (85; 77.4, 90.4)

#### 3.1.5 Associations Between Medication Beliefs and Key Factors in Medication Management

Table 4 presents the association between key factors—knowledge, capabilities and engagement and medication beliefs—following adjustment for age and gender covariates. Patients and family caregivers who were confident in their abilities had a statistically significant lower overall belief in medication harm (*β* = −1.23, [95% CI: −2.34, −0.13]). Patients and family caregivers who frequently asked HCPs about their medication 3–6 times (*β* = 2.72, [95% CI: 0.56, 4.88]) or more than 7 times (*β* = 1.88, SE 0.91, [95% CI: 0.06, 3.69]) had a statistically significant, higher overall belief in medication overuse, compared to those who never asked. Those who asked HCPS about their medications 1–2 times (*β* = 1.33, [95% CI: 0.23, 2.44]), 3–6 times (*β* = 1.57, [95% CI: 0.18, 2.97]) or more than 7 times (*β* = 2.65, [95% CI: 1.10, 4.20]) also had a statistically significant, higher overall belief in medication harm, compared to those who never asked.

**Table 4:** Unadjusted and adjusted coefficients for associations between key factors in medication management in patients and family caregivers and General‐overuse and General‐harm (*N* = 89).

	General‐overuse		General‐harm	
**Key factors in medication management**	Unadjusted *β* (95% CI)	Adjusted *β* (95% CI)	Unadjusted *β* (95% CI)	Unadjusted *β* (95% CI)
**Knowledge about medications**				
Knowledgeable versus not very knowledgeable	−0.72 (−1.77, 0.34)	0.75 (−2.08. 0.57)	−0.89 (−1.80, 0.01)	−0.39 (−1.42, 0.64)
**Confidence in the ability to keep an accurate medication list**				
Fairly confident/very confident versus not at all/somewhat confident	−0.43 (−1.60, 0.74)	−0.67 (−2.13, 0.78)	−1.49 (−2.39, −0.59)	−1.23 (−2.34, −0.13)*
**Engagement by patients and family caregivers**				
Asked HCPs about medications	—	—	—	—
Never	0.68 (0.63, 2.00)	0.96 (−0.57, 2.50)	0.98 (−0.08, 2.04)	1.33 (0.23, 2.44)*
1–2 times	2.59 (0.18, 3.12)	2.72 (0.56, 4.88)*	1.21 (−0.12, 2.54)	1.57 (0.18, 2.97)*
3–6 times	1.65 (0.92, 4.26)	1.88 (0.06, 3.69)*	2.55 (1.06, 4.05)*	2.65 (1.10, 4.20)*
More than 7 times				
**Time needed to engage**				
< 10 min	—	—	—	—
≥ 10 min	0.57 (−0.78, 1.92)	0.14 (−1.35, 1.62)	0.22 (−1.17, 1.62)	−0.24 (−1.62, 1.14)
Varying time	−0.71 (−2.2, 0.74)	−0.77 (−2.31, 0.77)	−0.06 (−1.23, 1.11)	−0.33 (−1.43, 0.77)
**Age**				
25–44 years	—	—	—	—
45–54 years	−0.31 (−1.65, 1.03)	−0.58 (−2.03, 0.87)	−0.72 (−1.83, 0.36)	−1.16 (−2.22, −0.01)
55–74 years	−0.18 (−1.66, 1.29)	−0.40 (−1.99, 1.19)	0.20 (−1.10, 1.50)	−0.01 (−1.40, 1.39)
**Gender**				
Female	—	—	—	—
Male	−0.21 (−1.35, 0.93)	−0.29 (−1.69, 1.10)	0.06 (−0.94, 1.06)	0.20 (−0.83, 1.23)

Abbreviations: *β* = beta, CI = confidence interval, min = minutes.

* Significant at *p* < 0.05 level.

## 4 Discussion

This study explored and compared some key SDM factors—perceived knowledge, perceived capabilities and engagement about medicines—in medication management. It examined how these factors may be associated with beliefs about medicines, within the context of SDM in the healthcare setting. This study found divergence between HCPs and patients and family caregivers, in their perceptions of patients and family caregivers' overall knowledge and capabilities. Patients and family caregivers often perceived their knowledge about medicines and confidence in their capabilities to manage medicines at higher levels than HCPs. This mismatch in perceptions can have significant implications for SDM. Patients and family caregivers, who consider themselves highly knowledgeable and capable, may reduce what they expect from their HCPs in the decision‐making process. This may lead to uninformed decision‐making, with insufficient consideration of treatment options and/or risk information. In contrast, HCPs' negative perceptions of knowledge and capability could dominate decision‐making, with inadequate consideration of patients' preferences and result in clinician‐led decisions [46].

Such self‐assessments by patients and family caregivers may not be accurate; previous research has demonstrated gaps in patients' knowledge about medicines and cognitive bias, where knowledge/abilities were overestimated [47, 48, 49]. However, research in patients with chronic disease and HCPs suggests that divergence between perceptions of knowledge and capabilities—such as reported between HCPs and patients and family caregivers in this study—could be addressed [50]. It examined the core values of HCP–patient interactions and found that a partnership approach could be encouraged. This would need HCPs to move from underestimating patients' abilities to acknowledging the value of patients' experiential knowledge as complimentary to their own competencies [50].

Patient and family caregivers' confidence in their capabilities to manage medication lists was associated with fewer concerns about medication harm. However, frequent engagement with HCPs about medications was associated with significant concerns about overuse and harm. Eliciting patients' health beliefs and concerns is critical to SDM [51], with negative medication beliefs associated with lower health literacy levels and poor adherence [33, 52, 53, 54, 55, 56, 57, 58]. Strategies to encourage disclosure of patients' perspectives include open‐ended questions (e.g., What concerns do you have/what risk concerns you most?), reflecting what has been said back to check interpretation, not speaking over patients/caregivers and reducing distractions or interruptions [51, 59]. Previous research in patient reporting of adverse drug reactions indicated that patients could often have elevated perceptions of symptom severity compared to HCPs [60]. In these circumstances, the type of engagement is key to effective HCP–patient risk communication. With insight into patients' medication beliefs, further discussions with HCPs can provide clear risk information in ways that foster patients' understanding, for example, simple written information, pictures and/or videos [51]. In this way, purposeful engagement by HCPs to identify whether risks are significant for patients can have mutual benefits. Patients and family caregivers can make informed decisions and unfounded concerns can be addressed, while HCPs can develop confidence and trust in their patients' medicines management.

These findings also support an expanded model of SDM, which has a strong focus on ensuring provider and patient skills [61]. This two‐tiered approach recognises that SDM requires multiple levels of health literacy skills (functional, communicative and critical) at different times [61]. Patients need cognitive and social skills, to express their personal preferences and critically evaluate health information [33, 62]. HCPs, in turn, need to access and appraise research evidence and engage in patient‐centred communication to support SDM [63]. SDM research has shown that such health literacy skills can be improved through education and training [61].

A central component of SDM is high‐quality patient–provider communication, which can improve adherence, build trust and provide beneficial health outcomes [53, 54]. While SDM is key to medical practice, HCPs have consistently identified limited time as a barrier to implementing SDM in daily practice [57, 58]. A noteworthy finding in our study indicated that SDM need not expand consultations—many patients and family caregivers and HCPs considered that short time intervals ( ≤ 10 min) were sufficient to engage with patients about medicines. This supports previous research, which found that applying SDM in routine clinical practice did not require longer consultation durations [60]. Research has identified the value of tools such as patient‐held medication lists (PHMLs) in facilitating conversations between HCPs and patients [42, 64]. It can be challenging for HCPs to align medication‐related decisions with patients' preferences [65]. However, skills training, such as increasing time management/listening skills or fostering explicit questioning about patients' goals, need not significantly increase consultation time [30]. Prioritising improvements to HCPs' communication skills could contribute to the sustained implementation of SDM in medication management.

## 5 Strengths and Limitations

This study included a range of viewpoints from a variety of HCPs, patients and family caregivers. Validated measures and qualitative findings were used to develop comprehensive surveys adapted for each group. However, individuals with distinct opinions about medication management may have been motivated to participate and may not be representative. Participants were not sampled for inclusion but self‐selected to complete the survey, which may have introduced bias in the responses observed. This self‐selection sampling and the small sample sizes associated with unstructured sampling have implications for the validity of the findings.

## 6 Conclusion

There has been an increased promotion of SDM as a fundamental component of healthcare globally, but its implementation into clinical practice is not universal. This study contributes to previous research by exploring key factors that can facilitate or inhibit SDM, with information from a variety of perspectives, HCPs, patients and family caregivers. This evidence is of interest in developing targeted interventions to support SDM in medication management.

## Author Contributions

**Bernadine O'Donovan:** formal analysis, writing – original draft, writing – review and editing. **Ciara Kirke:** writing – review and editing. **Muriel Pate:** writing – review and editing. **Sheena McHugh:** writing – review and editing. **Kathleen Bennett:** conceptualisation, methodology, writing – review and editing, funding acquisition, resources, supervision. **Caitríona Cahir:** conceptualisation, methodology, formal analysis, writing – review and editing, supervision.

## Ethics Statement

The questionnaire and methodology for this study were approved by the RCSI research ethics committee (REC: 212601097). All study procedures performed with human participants were in accordance with the ethical standards of the institutional and/or national research committee and with the 1964 Helsinki Declaration and its later amendments or comparable ethical standards.

## Consent

Informed consent was obtained from all survey respondents included in the study.

## Conflicts of Interest

Kathleen Bennett has received funding from Novartis and IQVIA for unrelated projects.

## Supporting information

Supporting_material.

## Data Availability

The authors have nothing to report.
