# Supplementary material for: A Quantitative Study Exploring and Comparing Key Factors in Medication Management in the Irish Healthcare Setting
Source: Health Expect. 2025 Apr 12;28(2):e70256. doi: 10.1111/hex.70256 (PMC11993810; doi:10.1111/hex.70256)
Supplement: Supplementary file 1 — Supporting_material. [file HEX-28-e70256-s001.docx]

# SURVEYS

# Patient survey

**The ‘Know Check Ask’ Medicine Safety campaign and Patient-held Medicine Lists: Your Views and Experiences**


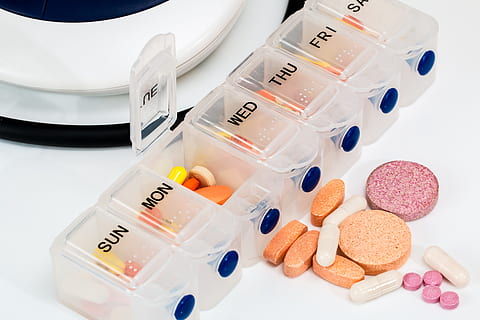


Thank you for participating in our survey. This survey has **4 sections (A-D)** and it will take no more than 15 minutes to complete all the sections. There are no right or wrong answers and we are interested in everyone’s opinions. Your responses will be confidential and we do not know who answered the survey. We ask that you answer questions as truthfully as possible so the results will be helpful in our research. If you do not want to answer any question then you can ignore it and continue on with the survey.

All the information that you will provide will be treated in confidence and in accordance with General Data Protection Regulations (GDPR). Once you have completed the questionnaire it can be returned in the pre-paid envelope (Freepost) to **The Division of Population Health Sciences, Beaux Lane House, St Stephens’ Green, Dublin 2.** Please answer **all** questions in Section A through to Section D by marking the boxes or filling in answers when requested to.

Section A: Managing medicines and patient held medicine lists

Q1. How knowledgeable are you about your medicines? (e.g. you know what medicines you are taking and why you are taking them)

| 🗆 Not knowledgeable | 🗆 Somewhat knowledgeable | 🗆 Knowledgeable | 🗆 Very knowledgeable |
| --- | --- | --- | --- |

Q2. What aids do you use to manage your medicines? *(you may select more than one option)*

|  |  |
| --- | --- |
| Pill box dispenser/organiser | 🗆 |
| Visual cues e.g., shape, colour of tablet | 🗆 |
| Pharmacy blister packs | 🗆 |
| Smartphone application | 🗆 |
| List of medicines provided by the GP | 🗆 |
| List of medicines provided by the pharmacist | 🗆 |
| Your own list of medicines | 🗆 |
| Photograph of the medicines list on your phone | 🗆 |
| Other    If **OTHER** selected, please specify | 🗆 |

Q3. In the past 12 months how many times have you asked your GP, pharmacist or nurse (known as healthcare professionals) about your medicines? *(please select one option)*

- Never
- 1-2 times
- 3-6 times
- 7 or more times

Q4. The following statements are about **medicines**, please rate your level of agreement with each statement. *(please tick one option per statement)*

|  | **Strongly disagree** | **Disagree** | **Neither agree or disagree** | **Agree** | **Strongly agree** |
| --- | --- | --- | --- | --- | --- |
| Doctors use too many medicines | 🗆 | 🗆 | 🗆 | 🗆 | 🗆 |
| People who take medicines should stop their treatment for a while every now and again | 🗆 | 🗆 | 🗆 | 🗆 | 🗆 |
| Most medicines are addictive | 🗆 | 🗆 | 🗆 | 🗆 | 🗆 |
| Natural remedies are safer than medicines | 🗆 | 🗆 | 🗆 | 🗆 | 🗆 |
| Medicines do more harm than good | 🗆 | 🗆 | 🗆 | 🗆 | 🗆 |
| All medicines are poison | 🗆 | 🗆 | 🗆 | 🗆 | 🗆 |
| Doctors place too much trust on medicines | 🗆 | 🗆 | 🗆 | 🗆 | 🗆 |
| If doctors had more time with patients they would prescribe fewer medicines | 🗆 | 🗆 | 🗆 | 🗆 | 🗆 |

Q5. The following statements are about **people’s lists of their medicines**, please rate your level of agreement with each statement. *(please tick one option per statement)*

|  | **Strongly disagree** | **Disagree** | **Neither agree or disagree** | **Agree** | **Strongly agree** |
| --- | --- | --- | --- | --- | --- |
| Keeping a list is important | 🗆 | 🗆 | 🗆 | 🗆 | 🗆 |
| Keeping a list would do more harm than good | 🗆 | 🗆 | 🗆 | 🗆 | 🗆 |
| I prefer to use my own list | 🗆 | 🗆 | 🗆 | 🗆 | 🗆 |
| Keeping a list is too much trouble for what I get out of it | 🗆 | 🗆 | 🗆 | 🗆 | 🗆 |
| Looking after my health depends on having a list | 🗆 | 🗆 | 🗆 | 🗆 | 🗆 |
| I have the skills to keep an accurate list | 🗆 | 🗆 | 🗆 | 🗆 | 🗆 |

Q6. In your opinion are people’s medicine lists reliable sources of information about their medicines? *(please tick one option)*

□ Yes (go to Q6b)

□ No (go to Q6c)

□ Don’t know (go to Q7)

Q6b. Why do you think people’s medicine lists **are** reliable sources of information?

Q6c. Why do you think people’s medicine lists **are not** reliable sources of information?

Q7. In your opinion which of the following details need to be included in people’s medicine lists? *(please select all that apply)*

|  |  |
| --- | --- |
| Name of medicine | 🗆 |
| Over the counter/non prescription medicines | 🗆 |
| Type of medicines e.g. tablets, creams | 🗆 |
| Why the medicine is being taken e.g. insulin for diabetes | 🗆 |
| Vitamins | 🗆 |
| Medicines taken for a short time/particular health problem | 🗆 |
| Dose/amount of medicine e.g. milligram (mg) | 🗆 |
| Repeat medicines that are taken regularily | 🗆 |
| Herbal medicinal products | 🗆 |
| Date that list was filled in | 🗆 |
| Other    If **OTHER** selected, please specify | 🗆 |

Q8. In your opinion, people mainly use their medicines lists to: *(please select all that apply)*

|  |  |
| --- | --- |
| Help talk with healthcare professionals about their medicines | 🗆 |
| Assist their memory | 🗆 |
| Help them when in/leaving hospital | 🗆 |
| Increase their awareness about the medicines they are taking | 🗆 |
| Help them to take medicines as prescribed for them | 🗆 |
| Assist them in emergencies | 🗆 |
| Help carers/family members who may need to know about a person’s medicines | 🗆 |
| Other    If **OTHER** selected, please specify | 🗆 |

| 🗆 Not at all confident | 🗆 Somewhat confident | 🗆 Fairly Confident | 🗆 Very Confident |
| --- | --- | --- | --- |

Q9. How confident are you that you would keep accurate medicine lists **without assistance** from your GP, pharmacist or nurse? *(please select one option)*

|  Not at all confident |  Somewhat confident |  Fairly Confident |  Very Confident |
| --- | --- | --- | --- |

Q10. How confident are you **to ask for assistance** from your GP, pharmacist or nurse in keeping accurate medicines lists? *(please select one option)*

Q11. In your opinion how long would it take during a consultation for GPs or pharmacists to engage with people about their medicines? *(please select one option)*

- Less than 5 minutes
- 5-10 minutes
- 10-15 minutes
- More than 15 minutes
- Other (please specify)

_______________________________

Section B: **Awareness and use of the HSE ‘Know Check Ask’ campaign**

Q12. Were you aware of the ‘**Know Check Ask’** campaign prior to this survey?

*(please select one option)*

- Yes
- No

Q13. Is the **‘Know Check ask’** message about knowing, checking and asking about your medicines something that you would support?

*(please select one option)*

- Yes
- No
- Don’t know

Q14. Have you used the HSE **‘My Medicines’ list** template **or** your own medicine list in the past 12 months? *(please select one option)*

- Used my own medicine list (go Q14b)
- Used HSE **‘My Medicines’ list** (go to Q14b)
- Used both my own list and HSE **‘My Medicines’ list** (go to Q14b)
- Did not use either of these (go to Q15)

Q14b. Please indicate if using the HSE **‘My Medicines’ list** template **or** your own medicines list has helped you in any of the following ways. *(please select all that apply)*

|  |  |
| --- | --- |
| Helped me to manage my medicines more effectively |  |
| Increased my awareness of what medicines I am taking |  |
| Not helped me in managing my medicines |  |
| Assisted me to take my medicines as prescribed for me |  |
| Created additional work for me |  |
| Increased my involvement in my healthcare |  |
| Reduced unused medicines |  |
| Helped me share information about my medicines when I am seeing a different doctor from usual |  |
| Helped me manage my repeat prescriptions |  |
| Other    If **OTHER** selected, please specify |  |

Q15. The following statements are about the [**'Know Check Ask'**](https://www.hse.ie/eng/about/who/nqpsd/patient-safety-programme/medication-safety/get-started-know-check-ask-for-people-who-use-medicines-and-their-families.html) message - about knowing, checking and asking about medicines. Please rate your level of agreement with the following statements

|  | **Strongly disagree** | **Disagree** | **Neither agree or disagree** | **Agree** | **Strongly agree** |
| --- | --- | --- | --- | --- | --- |
|  |  |  |  |  |  |
| It can help people who are taking multiple medicines |  |  |  |  |  |
| It is useful for people when there are changes in their healthcare e.g. admission or discharge from hospital |  |  |  |  |  |
| It can help people to talk to healthcare professionals about their medicines |  |  |  |  |  |
| It is useful for people in emergency situations |  |  |  |  |  |

Section C: **Future actions to promote the ‘Know Check Ask’ message**

Q16. In primary/community care which of the following healthcare professionals would be best placed to encourage people to keep and use medicine lists? *(you may select more than one)*

|  |  |
| --- | --- |
| GPs |  |
| Community pharmacists |  |
| Community nurses |  |
| Public health nurses |  |
| Other    If **OTHER** selected, please specify |  |

Q17. In a hospital setting which of the following healthcare professionals would be best placed to encourage people to keep and use medicine lists? *(you may select more than one)*

|  |  |
| --- | --- |
| Hospital doctors |  |
| Hospital pharmacists |  |
| Hospital nurses |  |
| Other    If **OTHER** selected, please specify |  |

|  | **Unlikely** | **Likely** | **Very likely** |
| --- | --- | --- | --- |
| My own paper based list |  |  |  |
| A list of my medicines printed for me by my GP |  |  |  |
| A list of my medicines printed for me by my pharmacist |  |  |  |
| A digital list on electronic device e.g. phone application |  |  |  |
| A compact list that would fit in my wallet/purse |  |  |  |

Q18. How likely is it that you would use any of the following types of medicine lists?

Q19. Which of the following do you think would encourage you to keep and use a medicines list? *(you may select more than one)*

|  |  |
| --- | --- |
| Hearing the message to keep a list on radio ads |  |
| My GP or pharmacist helping me to prepare a list of my medicines |  |
| My pharmacy adding a copy of the HSE **‘My Medicines’** list to the bag with prescriptions |  |
| Seeing the message to keep a list on social media |  |
| Message from GP or hospital to bring a list to appointments or admissions |  |
| A family member helping me to prepare a list |  |
| Seeing the message to keep a list in newspapers |  |
| Getting a blank list delivered to my home by post |  |
| Other    If **OTHER** selected, please specify |  |

Q20. Have you any further suggestions on how people can be encouraged to use medicine lists?

Section D: About You

| **Finally, we would like to ask some questions about you.The personal information you supply will be treated with strictest confidence.** |
| --- |

Q21. Please indicate your gender:

| □ Male  □ Female | □ Non binary/third gender  □ Prefer not to say |
| --- | --- |

Q22. Please indicate your age range:

| □ 18-24 years  □ 25-34 years  □ 35-44 years  □ 45-54 years  □ 55-64 years | □ 65-74 years  □ 75-84 years  □ 85 years or older  □ Prefer not to say |
| --- | --- |

Q23. Please indicate how many regularly prescribed medicines you currently use:

| □ 3 - 4  □ 5 - 9 | □ More than 10  □ Prefer not to say |
| --- | --- |

Q24. Please indicate if you have been diagnosed with or have any of the following health conditions (*tick all that apply*):

|  |  |
| --- | --- |
| Heart disease | 🗆 |
| Chronic lung disease such as chronic bronchitis or emphysema | 🗆 |
| Asthma | 🗆 |
| Arthritis including osteoarthritis or rheumatism | 🗆 |
| Osteoporosis | 🗆 |
| Cancer or malignant tumour including leukaemia or lymphoma but excluding minor skin cancers | 🗆 |
| Parkinson’s disease | 🗆 |
| Alzheimer’s disease | 🗆 |
| Any emotional, nervous or psychiatric problems such as depression or anxiety | 🗆 |
| Alcohol or substance abuse | 🗆 |
| Dementia, organic brain syndrome, senility | 🗆 |
| Serious memory impairment | 🗆 |
| Stomach ulcers | 🗆 |
| Varicose ulcers | 🗆 |
| Cirrhosis or serious liver damage | 🗆 |
| Prefer not to say | 🗆 |
| Other    If **OTHER** selected, please specify | 🗆 |

***Thank you for taking the time to complete this survey***

Should you have any questions or concerns about this survey, please contact the researcher.

B O’ Donovan

Email: [bernadineodonovan@rcsi.ie](mailto:bernadineodonovan@rcsi.ie)

Phone No: 087 3971060

Division of Population Health Sciences, Beaux Lane House, St Stephens’ Green, Dublin 2

# Caregivers’ survey

Section A: Managing medicines and patient held medicine lists

Q1. How knowledgeable are you about the medicines of the person you care for? (e.g. you know what medicines they are taking and why they are taking them)

| 🗆 Not knowledgeable | 🗆 Somewhat knowledgeable | 🗆 Knowledgeable | 🗆 Very knowledgeable |
| --- | --- | --- | --- |

Q2. What aids do you use to manage the medicines of the person you care for? *(you may select more than one option)*

|  |  |
| --- | --- |
| Pill box dispenser/organiser | 🗆 |
| Visual cues e.g., shape, colour of tablet | 🗆 |
| Pharmacy blister packs | 🗆 |
| Smartphone application | 🗆 |
| List of medicines provided by the GP | 🗆 |
| List of medicines provided by the pharmacist | 🗆 |
| Your own list of their medicines | 🗆 |
| Photograph of list of their medicines on your phone | 🗆 |
| Other    If **OTHER** selected, please specify | 🗆 |

Q3. In the past 12 months how many times have you asked your GP, pharmacist or nurse (known as healthcare professionals) about the medicines of the person you care for? *(please select one option)*

- Never
- 1-2 times
- 3-6 times
- 7 or more times

Q4. The following statements are about **medicines**, please rate your level of agreement with each statement. *(please tick one option per statement)*

|  | **Strongly disagree** | **Disagree** | **Neither agree or disagree** | **Agree** | **Strongly agree** |
| --- | --- | --- | --- | --- | --- |
| Doctors use too many medicines | 🗆 | 🗆 | 🗆 | 🗆 | 🗆 |
| People who take medicines should stop their treatment for a while every now and again | 🗆 | 🗆 | 🗆 | 🗆 | 🗆 |
| Most medicines are addictive | 🗆 | 🗆 | 🗆 | 🗆 | 🗆 |
| Natural remedies are safer than medicines | 🗆 | 🗆 | 🗆 | 🗆 | 🗆 |
| Medicines do more harm than good | 🗆 | 🗆 | 🗆 | 🗆 | 🗆 |
| All medicines are poison | 🗆 | 🗆 | 🗆 | 🗆 | 🗆 |
| Doctors place too much trust on medicines | 🗆 | 🗆 | 🗆 | 🗆 | 🗆 |
| If doctors had more time with patients they would prescribe fewer medicines | 🗆 | 🗆 | 🗆 | 🗆 | 🗆 |

Q5. The following statements are about **carers' lists of the medicines of the person they care for,** please rate your level of agreement with each statement. *(please tick one option per statement)*

|  | **Strongly disagree** | **Disagree** | **Neither agree or disagree** | **Agree** | **Strongly agree** |
| --- | --- | --- | --- | --- | --- |
| Keeping a list is important | 🗆 | 🗆 | 🗆 | 🗆 | 🗆 |
| Keeping a list would do more harm than good | 🗆 | 🗆 | 🗆 | 🗆 | 🗆 |
| I prefer to use my own list | 🗆 | 🗆 | 🗆 | 🗆 | 🗆 |
| Keeping a list is too much trouble for what I get out of it | 🗆 | 🗆 | 🗆 | 🗆 | 🗆 |
| Looking after the health of the person I care for depends on having a list | 🗆 | 🗆 | 🗆 | 🗆 | 🗆 |
| I have the skills to keep an accurate list | 🗆 | 🗆 | 🗆 | 🗆 | 🗆 |

Q6. In your opinion are carers' lists, of the medicines of the person they care for, reliable sources of information about their medicines? *(please tick one option)*

□ Yes (go to Q6b)

□ No (go to Q6c)

□ Don’t know (go to Q7)

Q6b. Why do you think carers' lists, of the medicines of the person they care for, **are** reliable sources of information?

Q6c. Why do you think carers' lists of the medicines of the person they care for, **are not** reliable sources of information?

Q7. In your opinion which of the following details need to be included in carers' lists of the medicines of the person they care for? *(please select all that apply)*

|  |  |
| --- | --- |
| Name of medicine | 🗆 |
| Over the counter/non prescription medicines | 🗆 |
| Type of medicines e.g. tablets, creams | 🗆 |
| Why the medicine is being taken e.g. insulin for diabetes | 🗆 |
| Vitamins | 🗆 |
| Medicines taken for a short time/particular health problem | 🗆 |
| Dose/amount of medicine e.g. milligram (mg) | 🗆 |
| Repeat medicines that are taken regularly | 🗆 |
| Herbal medicinal products | 🗆 |
| Date that list was filled in | 🗆 |
| Other    If **OTHER** selected, please specify | 🗆 |

|  |  |
| --- | --- |
| Help talk with healthcare professionals about medicines | 🗆 |
| Assist their memory | 🗆 |
| Help them when the person they care for is in/leaving hospital | 🗆 |
| Increase their awareness about the medicines that are being taken | 🗆 |
| Check medicines are being taken as prescribed | 🗆 |
| Assist them in emergencies | 🗆 |
| Help family members who may need to know about a person’s medicines | 🗆 |
| Other    If **OTHER** selected, please specify | 🗆 |

Q8. In your experience, carers mainly use their lists, of the medicines of the person they care for to: *(please select all that apply)*

| 🗆 Not at all confident | 🗆 Somewhat confident | 🗆 Fairly Confident | 🗆 Very Confident |
| --- | --- | --- | --- |

Q9. How confident are you, as a carer, that you would keep accurate medicine lists **without assistance** from your GP, pharmacist or nurse? *(please select one option)*

|  Not at all confident |  Somewhat confident |  Fairly Confident |  Very Confident |
| --- | --- | --- | --- |

Q10. How confident are you, as a carer, **to ask for assistance** from your GP, pharmacist or nurse in keeping accurate medicines lists? *(please select one option)*

Q11. How long do you think it would take during a consultation for GPs or pharmacists to engage with carers about medicines? *(please select one option)*

- Less than 5 minutes
- 5-10 minutes
- 10-15 minutes
- More than 15 minutes
- Other (please specify)

_______________________________

Section B: **Awareness and use of the HSE ‘Know Check Ask’ campaign**

Q12. Were you aware of the ‘**Know Check Ask’** campaign prior to this survey?

*(please select one option)*

- Yes
- No

Q13. Is the **‘Know Check ask’** message about knowing, checking and asking about your medicines something that you would support?

*(please select one option)*

- Yes
- No
- Don’t know

Q14. Have you used the HSE **‘My Medicines’ list** template **or** your own medicine list in the past 12 months? *(please select one option)*

- Used my own medicine list (go Q14b)
- Used HSE **‘My Medicines’ list** (go to Q14b)
- Used both my own list and HSE **‘My Medicines’ list** (go to Q14b)
- Did not use either of these (go to Q15)

Q14b. Please indicate if using the HSE **‘My Medicines’ list** template **or** your own medicines list has helped you in any of the following ways. *(please select all that apply)*

|  |  |
| --- | --- |
| Helped me to manage my medicines more effectively |  |
| Increased my awareness of what medicines I am taking |  |
| Not helped me in managing my medicines |  |
| Assisted me to take my medicines as prescribed for me |  |
| Created additional work for me |  |
| Increased my involvement in my healthcare |  |
| Reduced unused medicines |  |
| Helped me share information about my medicines when I am seeing a different doctor from usual |  |
| Helped me manage my repeat prescriptions |  |
| Other    If **OTHER** selected, please specify |  |

Q15. The following statements are about the [**'Know Check Ask'**](https://www.hse.ie/eng/about/who/nqpsd/patient-safety-programme/medication-safety/get-started-know-check-ask-for-people-who-use-medicines-and-their-families.html) message - about knowing, checking and asking about medicines. Please rate your level of agreement with the following statements

|  | **Strongly disagree** | **Disagree** | **Neither agree or disagree** | **Agree** | **Strongly agree** |
| --- | --- | --- | --- | --- | --- |
|  |  |  |  |  |  |
| It can help people who are taking multiple medicines |  |  |  |  |  |
| It is useful for people when there are changes in their healthcare e.g. admission or discharge from hospital |  |  |  |  |  |
| It can help people to talk to healthcare professionals about their medicines |  |  |  |  |  |
| It is useful for people in emergency situations |  |  |  |  |  |

Section C: **Future actions to promote the ‘Know Check Ask’ message**

Q16. In primary/community care which of the following healthcare professionals would be best placed to encourage carers to keep and use lists of the medicines of the person they care for? *(you may select more than one)*

|  |  |
| --- | --- |
| GPs |  |
| Community pharmacists |  |
| Community nurses |  |
| Public health nurses |  |
| Other    If **OTHER** selected, please specify |  |

Q17. In a hospital setting which of the following healthcare professionals would be best placed to encourage carers to keep and use lists of the medicines of the person they care for? *(you may select more than one)*

|  |  |
| --- | --- |
| Hospital doctors |  |
| Hospital pharmacists |  |
| Hospital nurses |  |
| Other    If **OTHER** selected, please specify |  |

|  | **Unlikely** | **Likely** | **Very likely** |
| --- | --- | --- | --- |
| My own paper based list |  |  |  |
| A list of my medicines printed for me by my GP |  |  |  |
| A list of my medicines printed for me by my pharmacist |  |  |  |
| A digital list on electronic device e.g. phone application |  |  |  |
| A compact list that would fit in my wallet/purse |  |  |  |

Q18. How likely is it that you would use any of the following types of medicine lists?

Q19. Which of the following do you think would encourage you, as a carer, to keep and use a list of the medicines of the person you care for? *(you may select more than one)*

|  |  |
| --- | --- |
| Hearing the message to keep a list on radio ads |  |
| My GP or pharmacist helping me to prepare a list of my medicines |  |
| My pharmacy adding a copy of the HSE **‘My Medicines’** list to the bag with prescriptions |  |
| Seeing the message to keep a list on social media |  |
| Message from GP or hospital to bring a list to appointments or admissions |  |
| A family member helping me to prepare a list |  |
| Seeing the message to keep a list in newspapers |  |
| Getting a blank list delivered to my home by post |  |
| Other    If **OTHER** selected, please specify |  |

Q20. Have you any further suggestions on how carers can be encouraged to use medicine lists?

Section D: About You

| **Finally, we would like to ask some questions about you. The personal information you supply will be treated with strictest confidence.** |
| --- |

Q21. Please indicate your gender:

| □Male  □Female | □Non-binary/third gender  □Prefer not to say |
| --- | --- |

Q22. Please indicate your age range:

| □ 18-24 years  □ 25-34 years  □ 35-44 years  □ 45-54 years | □ 55-64 years  □ 65-74 years  □ 75-84 years  □ 85 years or older  □ Prefer not to say |
| --- | --- |

Q23. Please indicate how many regularly prescribed medicines are currently used by the person you care for:

| □ 3 - 4  □ 5 - 9 | □ More than 10  □ Prefer not to say |
| --- | --- |

Q24. Please indicate if the person that you care for has been diagnosed with or have any of the following health conditions (tick all that apply):

|  |  |
| --- | --- |
| Heart disease | 🗆 |
| Chronic lung disease such as chronic bronchitis or emphysema | 🗆 |
| Asthma | 🗆 |
| Arthritis including osteoarthritis or rheumatism | 🗆 |
| Osteoporosis | 🗆 |
| Cancer or malignant tumour including leukaemia or lymphoma but excluding minor skin cancers | 🗆 |
| Parkinson’s disease | 🗆 |
| Alzheimer’s disease | 🗆 |
| Any emotional, nervous or psychiatric problems such as depression or anxiety | 🗆 |
| Alcohol or substance abuse | 🗆 |
| Dementia, organic brain syndrome, senility | 🗆 |
| Serious memory impairment | 🗆 |
| Stomach ulcers | 🗆 |
| Varicose ulcers | 🗆 |
| Cirrhosis or serious liver damage | 🗆 |
| Prefer not to say | 🗆 |
| Other    If **OTHER** selected, please specify | 🗆 |

***Thank you for taking the time to complete this survey***

Should you have any questions or concerns about this survey, please contact the researcher.

B O’Donovan

Email: bernadineodonovan@rcsi.ie

Phone No.:0873971060

Division of Population Health Sciences, Beaux Lane House, St Stephens’ Green, Dublin 2

# HCP survey (online)

# Community pharmacists

Instructions

Thank you for participating in our survey. This survey has 4 sections (A-D) and it will take no more than 15 minutes to complete all the sections. There are no right or wrong answers and we are interested in everybody’s opinions.

Your replies will be confidential and we ask that you answer the questions as candidly as possible in order for the results to be of value in facilitating further implementation of the **'Know, Check, Ask'** campaign.

All the information that you will provide will be treated in confidence and in accordance with General Data Protection Regulations (GDPR).

Section A Managing medication and patient held medication lists

Q1 How knowledgeable are people about their medications? (e.g. know what medications they are taking and why they are taking them)  please select one option)

- Not knowledgeable
- Somewhat knowledgeable
- Knowledgable
- Very knowledgeable

|  | Strongly disagree | Disagree | Neither agree or disagree | Agree | Strongly agree |
| --- | --- | --- | --- | --- | --- |
| A person who keeps a medicines list is less likely to experience an adverse event |  |  |  |  |  |
| A person who keeps a medicines list is more adherent |  |  |  |  |  |
| A person who keeps a medicines list is better able to communicate at transitions of care |  |  |  |  |  |
| It is helpful to me when a person uses a list |  |  |  |  |  |
| It is helpful to the person when they use a list |  |  |  |  |  |
| I have time to engage with people/carers about their medication list |  |  |  |  |  |
| I have time to answer any questions people/carers have about their medication |  |  |  |  |  |

Q2 The following statements are about patient held medication lists. Please rate your level of agreement with each statement (please tick one option per statement)

Q3 The following statements are about your day-to-day practice please rate how often you engage in the following activities (please tick one option per statement)

|  | Never | Rarely | Sometimes | Often | Always |
| --- | --- | --- | --- | --- | --- |
| I engage in shared decision making with people about their medicines |  |  |  |  |  |
| I help people understand their medicines and address any questions they have |  |  |  |  |  |
| I encourage people to keep a medicines list |  |  |  |  |  |
| I ensure that a person's medicines list is accurate |  |  |  |  |  |
| I use a person’s medicines list to update the pharmacy record of their medicines |  |  |  |  |  |
| I give people a copy of the medicines list we have in the pharmacy for them |  |  |  |  |  |

Q4 What aids are you aware of that people or their carers use to manage their medications? (please select all that apply)

- Pill box dispenser/organiser filled by patient/carer
- Visual cues e.g. shape, colour of tablets
- Pharmacy blister packs
- Smartphone application
- GP list of medications
- Pharmacy list of medications
- Their own medicine list
- Photograph of list on phone
- Don't know
- Other (please specify) ________________________________________________

Q5 How often do you ask people or their carers during a consultation if they keep/have a medication list? (please select one option)

- Never
- Sometimes
- Often
- Always

Q6 How often in the pharmacy do people or their carers share their medicines list with you? (please select one option)

- Never
- Sometimes
- Often
- Always

Q7 If people or their carers share their medicines list with me I use it: (please select all that apply)

- To review their medicines
- To check their adherence
- To identify medication errors
- To check their understanding of their medicines
- Other (please specify) ________________________________________________

Q8 How confident are you that people/carers would keep accurate medication lists? (please select one option)

- Not at all confident
- Somewhat confident
- Fairly confident
- Very confident

Q9 In your opinion which of the following details need to be included in people's medication lists? (please select all that apply)

- Name of prescribed medication
- Type of medication
- Quantity of drugs
- Dose of drugs
- Strength of drug
- Indication for use
- Whether medication is acute or chronic
- Over the counter medications
- Vitamins
- Herbal medicinal products
- When they are taken
- Date form was filled
- Other (please specify) ________________________________________________

Q10 In your experience how frequently does a person's medication list need to be reviewed by them? (please select one option)

- Weekly
- Monthly
- Every 6 months
- Every year
- Only when there is a change in medications
- Other (please specify) ________________________________________________

Q11 The following statements are about people that may have difficulties keeping accurate medication lists. Please indicate how often you think the following types of people would have difficulty in keeping accurate medicines lists without support from family/carers (please tick one option per statement)

|  | Never | Sometimes | Often | Always |
| --- | --- | --- | --- | --- |
| Those who are older (aged 65+ years) |  |  |  |  |
| Those with cognitive impairments/dementia |  |  |  |  |
| Those with low literacy skills |  |  |  |  |
| Those on multiple medications |  |  |  |  |

Q12 Which of the following types of patient held medication lists would you prefer to be used by people/carers? (please tick one option)

- Paper based lists
- Digital lists e.g. phone apps
- Both paper and digital lists
- Neither of these

Q13 Should pharmacists always check the accuracy of people's medication lists? (please tick one option)

- Yes
- No
- Don't know

Q14 Which of the following options would you use to check the accuracy of a person’s medication list? (please select all that apply)

- Their regular GP
- Hospital pharmacist
- Your pharmacy records
- Hospital specialist
- Hospital nurse
- Other (please specify) ________________________________________________

Q15 From your own experience, do you consider that patient held medication lists are reliable sources of information about medication? (please tick one option)

- Never
- Sometimes
- Often
- Always

Q16 From your own experience, what is the primary use of people’s medication lists? (please select all that apply)

- Communication tool
- Memory aid for patients
- Compliance/adherence tool
- Complete record of medication
- Other (please specify) ________________________________________________

Q17 During a routine interaction in the pharmacy, how long would it take, on average, to engage with a person/carer about their medication? (please tick one option)

- Less than 5 minutes
- 5-10 minutes
- 10-15 minutes
- More than 15 minutes
- Other (please specify) ________________________________________________

Q18 Which of the following information sources should be reviewed by HCPs to form an accurate medication history at a transition of care? (please select all that apply)

- Community pharmacy record
- Hospital pharmacist
- Hospital discharge prescription/letter
- GP referral letter
- GP practice records
- Patient's verbal account
- Patient held medication list
- Nursing home list
- Patient’s own drugs
- Consultant letter
- Other (please specify) ________________________________________________

Q19  The following information sources can be used to form an accurate medication history at care transitions. Please rate all of them on a scale of 1-10, where 1 is the least reliable source and 10 is the most reliable source.

|  | 1 = Least reliable source | Reliability scale | 10 = Most reliable source |
| --- | --- | --- | --- |

|  | 1 | 2 | 3 | 4 | 5 | 6 | 6 | 7 | 8 | 9 | 10 |
| --- | --- | --- | --- | --- | --- | --- | --- | --- | --- | --- | --- |

| Community pharmacy record | 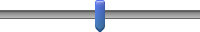 |
| --- | --- |
| Hospital pharmacist | 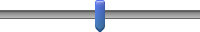 |
| Hospital discharge prescription/letter | 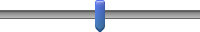 |
| GP referral letter | 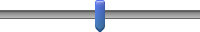 |
| GP practice records | 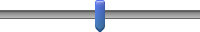 |
| Patient's verbal account | 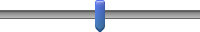 |
| Patient held medication list | 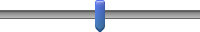 |
| Nursing home list | 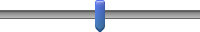 |
| Patient’s own drugs | 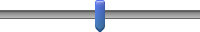 |
| Consultant letter | 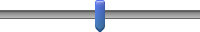 |

**Section B Awareness and use of the 'Know, Check, Ask' campaign**

Q20 Were you aware of the '**Know Check Ask'** campaign/message prior to this survey?

- Yes
- No
- Don't know

Q21 Given what you know of the **'Know Check Ask'** campaign, is the **'Know Check Ask'** message something that you would support?

- Yes
- No
- Don't know

Q22 Have you used any of the ‘**Know Check Ask’** resources that are available to HCPs?

- Yes
- No
- Don't know

Q22b Which of the following resources did you use to promote the **'Know Check Ask'** message and encourage the use of patient held medication lists? (select all that apply)

- Informational videos
- Printed 'My Medicines' lists
- Information leaflets
- Posters
- Information on www.safermeds.ie
- Other (please specify) ________________________________________________

Q22c How useful were the resources that you used on a scale of 1- 10, where 1 is the least useful resource and 10 is the most useful resource?

|  | 1 = Least useful resource | Usefulness scale | 10 = Most useful resource |
| --- | --- | --- | --- |

|  | 1 | 2 | 3 | 4 | 5 | 6 | 6 | 7 | 8 | 9 | 10 |
| --- | --- | --- | --- | --- | --- | --- | --- | --- | --- | --- | --- |

| Informational videos | 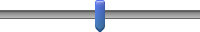 |
| --- | --- |
| Printed 'My Medicines' lists | 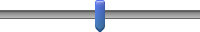 |
| Information leaflets | 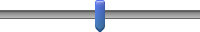 |
| Posters | 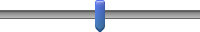 |
| Information on www.safermeds.ie | 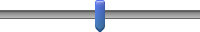 |
| Other (please specify) | 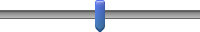 |

Q23 Has your pharmacy promoted the ‘**Know Check Ask’** message in the past 12 months?

- Yes
- No
- Don't know

Q24 Have any of the people that use your pharmacy heard of the **'Know Check Ask'** message and are now using a medicine list to communicate with you about their medicines?

- Yes
- No
- Don't know

Q25 Encouraging people to use medications lists or promoting the **'Know Check Ask'** message would: (please select all that apply)

- Help me to identify medication issues
- Not assist me in my pharmacy
- Prevent medication errors
- Increase my workload
- Empower people
- Reduce unused/wasted medications
- Improve people’s adherence
- Create additional burden for people
- Increase people’s awareness of their medications
- Assist those using multiple medications
- Help patient safety at transitions of care
- Other (please specify) ________________________________________________

**Section C Future actions to promote the ‘Know Check Ask’ message**

Q26 Which of the following HCPs do you think would be best placed to promote the ‘**Know Check Ask’** message and encourage use of patient held medication lists? (you may select more than one option)

- GPs
- Hospital doctors
- Community pharmacists
- Hospital pharmacists
- Hospital nurses
- Practice nurses
- Public health nurses
- Other (please specify) ________________________________________________

Q27 Given what you know about the current ‘**Know Check Ask’** resources, do any additional resources need to be made available to HCPs to help them promote the ‘**Know Check Ask’** message?

- Yes (*Skip To: Q27b)*
- No *(Skip To: Q28)*
- Don't know *(Skip To: Q28)*

Q27b In your opinion what additional resources need to be made available to HCPs?

________________________________________________________________

________________________________________________________________

________________________________________________________________

________________________________________________________________

________________________________________________________________

Q28 How could the HSE help community pharmacists to promote the **'Know Check Ask'** message and use of medicine lists?

________________________________________________________________

________________________________________________________________

________________________________________________________________

________________________________________________________________

________________________________________________________________

**Section D About You**

Please Note: The personal information you supply will be treated with strictest confidence

Q29 Please indicate your gender:

- Male
- Female
- Non-binary / third gender
- Prefer not to say

Q30 Please indicate your age range:

- 22-34 years
- 35-44 years
- 45-54 years
- 55-64 years
- 65+ years
- Prefer not to say

Q31 Please indicate the number of years since you qualified:

- Less than 5 years
- 5-10 years
- 11-15 years
- 16-20 years
- 21-25 years
- More than 25 years
- Prefer not to say

END OF SURVEY

**_________________________________________________________________________**
